# Seneca Valley Virus 2C and 3C^pro^ Induce Apoptosis via Mitochondrion-Mediated Intrinsic Pathway

**DOI:** 10.3389/fmicb.2019.01202

**Published:** 2019-05-29

**Authors:** Tingting Liu, Xiangmin Li, Mengge Wu, Liuxing Qin, Huanchun Chen, Ping Qian

**Affiliations:** ^1^State Key Laboratory of Agricultural Microbiology, College of Veterinary Medicine, State Key Laboratory of Agricultural Microbiology, Huazhong Agricultural University, Wuhan, China; ^2^Laboratory of Animal Virology, College of Veterinary Medicine, Huazhong Agricultural University, Wuhan, China; ^3^Key Laboratory of Development of Veterinary Diagnostic Products, Ministry of Agriculture, Wuhan, China; ^4^Key Laboratory of Preventive Veterinary Medicine in Hubei Province, The Cooperative Innovation Center for Sustainable Pig Production, Wuhan, China

**Keywords:** seneca valley virus (SVV), 2C, 3C^pro^, apoptosis, caspase-3

## Abstract

Seneca Valley virus (SVV) is the only member of the genus *Senecavirus* of the *Picornaviridae* family. SVV can selectively infect and lyse tumor cells with neuroendocrine features and is used as an oncolytic virus for treating small-cell lung cancers. However, the detailed mechanism underlying SVV-mediated destruction of tumor cells remains unclear. In this study, we found that SVV can increase the proportion of apoptotic 293T cells in a dose- and time-dependent manner. SVV-induced apoptosis was initiated via extrinsic and intrinsic pathways through activation of caspase-3, the activity of which could be attenuated by a pan-caspase inhibitor (Z-VAD-FMK). We confirmed that SVV 2C and 3C^pro^ play critical roles in SVV-induced apoptosis. The SVV 2C protein was located solely in the mitochondria and activated caspase-3 to induce apoptosis. SVV 3C^pro^ induced apoptosis through its protease activity, which was accompanied by release of cytochrome C into the cytoplasm, but did not directly cleave PARP1.

## Introduction

Seneca Valley virus (SVV, also called Senecavirus A), the only member of the genus *Senecavirus* of family *Picornaviridae*, is a positive-sense, non-enveloped virus with a single-stranded RNA genome (Hales et al., 2008). SVV infection can cause vesicular disease and is associated with porcine idiopathic vesicular disease and transient epidemic neonatal losses, especially among herds with SVV-infected sows (Pasma et al., 2008; Canning et al., 2016; Qian et al., 2016; Leme et al., 2017). SVV is used for the treatment of neuroendocrine tumors in humans because it has potent cytolytic activity and high selectivity for tumor cell lines of neuroendocrine origin, such as small-cell lung cancers and solid pediatric cancer cells (Seshidhar et al., 2007).

The full-length SVV genome is approximately 7.3 kb in length and consists of a 5′-untranslated region (UTR), a long open-reading frame (ORF) and a polyadenylated 3′-UTR. The ORF is translated into a large polyprotein which is cleaved into four structural proteins (VP4-VP2-VP3-VP1) and eight non-structural proteins (L-2A-2B-2C-3A-3B-3C-3D) by viral and host proteases (Hales et al., 2008). The viral non-structural proteins are responsible for viral replication (Hales et al., 2008). Among these non-structural proteins, 2C is one of the most conserved (Norder et al., 2011), and 3C^pro^ can participate in cleavage of the viral polyprotein during replication of SVV (Hales et al., 2008). The 2C protein is predicted to possess helicase activity due to the presence of conserved motifs (A and B) found in NTP-binding proteins, as well as the C motif, which is a typical feature of members of SF3 helicases (Gorbalenya and Koonin, 1993; Hales et al., 2008; Norder et al., 2011). In addition, SVV 3C^pro^ has been confirmed to cleave various host proteins through its enzymatic activity (Qian et al., 2017). However, the functions of most SVV proteins have not been studied and the mechanisms of viral pathogenesis remain unclear.

Apoptosis is a process of programmed cell death and is critical for normal development of cells and tissues and for maintenance of tissue homeostasis. Abnormalities in apoptosis are involved in cancer and neurodegenerative disease (Carson and Ribeiro, 1993; Friedlander, 2003). Apoptosis can be initiated through extrinsic and intrinsic pathways, and is primarily executed by a family of cysteine proteases known as caspases (Hengartner, 2000; Riedl and Shi, 2004). The extrinsic pathway is triggered by extracellular stimuli and is regulated by membrane death receptors, while the intrinsic pathway is triggered by cellular stress and is regulated by mitochondrion-associated proteins that cause release of cytochrome C into the cytoplasm (Brenner and Kroemer, 2000; Reed, 2000; Acehan et al., 2002). Released cytochrome C can bind Apaf-1, inducing its oligomerization and causing caspase-9 and caspase-3 activation. Both pathways eventually result in activation of caspase-3, which subsequently cleaves many substrates such as poly (ADP-ribose) polymerase (PARP), resulting in nuclear fragmentation and eventually leading to apoptosis (Budihardjo et al., 1999).

Oncolytic virotherapy has been emerging as a promising therapeutic strategy for cancer. Oncolytic viruses selectively infect, replicate within, and/or kill cancer cells using various approaches, such as re-establishing anti-tumor immune surveillance, direct receptor stimulation, or induction of tumor apoptosis (Alan et al., 2011; Drakes and Stiff, 2014; Suzuki et al., 2016). Previous studies have confirmed that oncolytic poxviruses may promote cell death by inducing apoptosis and programmed necrosis in cancer cell lines. Coxsackievirus A21 was identified as a novel oncolytic virus and induced immunogenic apoptosis in bladder cancer cell lines, and caprine herpesvirus type 1 induced apoptosis in several biological systems (Annels et al., 2018; Hu et al., 2018; Montagnaro et al., 2018).

Oncolytic viruses can replicate specifically within tumor cells and induce effects leading to cell lysis and apoptosis. Thus, the question of whether SVV’s utility as an oncolytic virus were related to apoptosis was an important one. It has been reported that SVV can induce apoptosis, but the detailed mechanisms underlying this process remain to be elucidated (Yu et al., 2011). In this study, we investigated how the SVV-HB strain induces apoptosis as well as the apoptotic pathways involved in this process in different cell lines. We found SVV 2C and 3C^pro^ induced apoptosis by activating caspase-3. These results may help better understand the pathogenesis of SVV, and provide novel insights to explain the oncolytic mechanism employed by SVV.

## Materials and Methods

### Cells and Viruses

Baby hamster kidney (BHK-21) cells and human embryonic kidney (293T) cells were grown at 37°C in Dulbecco’s modified essential medium (Invitrogen, Carlsbad, CA, United States) supplemented with 10% (v/v) fetal bovine serum (FBS, Invitrogen, Grand Island, NY, United States). H1299 cells and SW620 cells were grown at 37°C in RPMI-1640 (Invitrogen, Carlsbad, CA, United States) containing 10% FBS in a humidified 5% CO_2_ incubator. SVV HB-CH-2016 strains were propagated and the viral titer was determined in BHK-21 cells by plaque assay (Fan et al., 2016; Qian et al., 2016). Briefly, the supernatant was inoculated onto the monolayer of BHK-21 cells, and the cytopathic effects (CPE) was observed daily under a microscope. Culture supernatants were harvested and re-inoculated onto fresh BHK-21 cells until the typical CPE of SVV appeared. The virus was purified by CsCl-gradient ultracentrifugation (1.33 *g*/ml) (Seshidhar et al., 2007; Cao et al., 2018).

### Antibodies

Mouse monoclonal antibody against HA-tag and horseradish peroxidase-conjugated (HRP)-conjugated goat anti-rabbit IgG (H+L) secondary antibodies were purchased from Medical & Biological Laboratories Co., Ltd., (MBL, Japan). Rabbit anti-PARP1 polyclonal antibody (13371-1-AP), rabbit anti-Bax polyclonal antibody (50599-2-Ig), rabbit anti-Tom20 polyclonal antibody (11802-1-AP), mouse anti-cytochrome C monoclonal antibody (66264-1-AP), mouse anti-green fluorescent protein monoclonal antibody (66002-1-Ig), mouse anti-alpha tubulin monoclonal antibody (66031-1-Ig), and mouse anti-glyceraldehyde-3-phosphate dehydrogenase (GAPDH) monoclonal antibody (60004-1-Ig) were purchased from Proteintech Group Inc., (China). HRP-conjugated goat anti-mouse IgG (H+L) secondary antibody was obtained from Boster Bioengineering Ltd (China). Rabbit polyclonal anti-Bak were purchased from ABclone (Wuhan, China). Rabbit anti-VP1 polyclonal antibody was generated in our laboratory.

### Co-immunoprecipitation (Co-IP) Analysis and Western Blotting

293T cells were cotransfected with the indicated plasmids, then 24 h later the cells were harvested and lysed in lysis buffer [50 mM HEPES, 150 mM NaCl, 1 mM EDTA, 1% (v/v) NP40, and protease inhibitors] (Roche, United Kingdom). The samples were centrifuged for 10 min at 4°C to remove cellular debris. Cell lysates were incubated with the indicated antibody at 4°C overnight in a rolling incubator. Protein A/G-agarose beads (Santa Cruz Biotechnology, Inc.) were added to the lysates and incubated for another 4 h. The agarose beads were washed five times with cold lysis buffer. Bound proteins were boiled and analyzed by western blotting with the appropriate antibody. For western blotting, cells were harvested at the indicated time points and treated with lysis buffer. The protein concentrations of whole-cell lysates were measured using a bicinchoninic acid protein assay kit (Thermo Scientific). Equal amounts of proteins were electrophoresed on 12% sodium dodecyl sulfate polyacrylamide gels and transferred onto polyvinylidene fluoride membranes (Roche, United Kingdom). The membranes were blocked with 5% (v/v) non-fat milk in Tris-buffered saline containing 5% (v/v) Tween-20 (DGBio, Beijing, China) for 4 h at room temperature (RT). The membranes were subsequently incubated with diluted primary antibodies at RT or at 4°C for 2 h or 16 h, respectively. HRP-conjugated anti-rabbit or anti-mouse IgGs were used as secondary antibodies. An enhanced chemiluminescent substrate was used for detection (Thermo Scientific, United States). All immunoblot images were obtained using a Bio-Rad ChemiDoc XRS+ instrument and image software. Expression of GAPDH or alpha tubulin was assessed using anti-GAPDH or anti-alpha tubulin monoclonal antibodies and used as internal references.

### Confocal Microscopy

293T cells were seeded on coverslips in 24-well plates for 16 h and transfected with the indicated plasmids. After 24 h, the cells were fixed with 4% paraformaldehyde for 20 min and permeabilized with 0.5% Triton X-100 (Genview) at RT for 10 min. The cells were washed three times with phosphate-buffered saline (PBS) and blocked with PBS containing 5% (w/v) bovine serum albumin for 1 h at RT. The cell were then incubated with appropriate primary antibodies for 1 h at RT. After washing three times with PBS, cells were incubated with Alexa Fluor 488- and Alexa Fluor 555-conjugated secondary antibodies (diluted 1:1,000) for 1 h at RT. After washing three times with PBS, the cells were stained with 4′,6-diamidino-2-phenylindole (DAPI, Sigma) for 5 min. Finally, the cells were washed again and observed using a confocal laser scanning microscope (LSM 510 Meta; Carl Zeiss).

### Detection of Caspase-9, Caspase-8, and Caspase-3 Activity

Caspase-9, caspase-8, and caspase-3 activities were determined using a caspase assay kit according to the manufacturer’s instructions (Beyotime, China). Briefly, cells were lysed and protein concentrations were determined by the Bradford method (Beyotime, China). Caspase-3, caspase-8 and caspase-9 activities were measured using their respective substrate peptides Ac-DEVD-ρNA, Ac-IETD-ρNA and Ac-LEHD-ρNA. A mixture of 80 μl of detection buffer, 10 μl of sample and 10 μl of either Ac-DEVD-ρNA, Ac-IETD-ρNA or Ac-LEHD-ρNA was incubated at 37°C for 6 h. Release of ρ-nitroanilide (ρNA) was quantitated by measuring absorbance at 405 nm with a microplate reader. Caspase-3, caspase-8 and caspase-9 activities were calculated based on a standard curve. Blank values were subtracted, and increases in caspase-3 activities were expressed as fold increase and calculated based on activities measured from the control group. All experiments were performed in triplicate.

### Mitochondrial and Cytosolic Fractionation

Mitochondrial and cytosolic protein fractions were prepared using a Cell Mitochondria Isolation Kit according to a standard procedure (Beyotime Institute of Biotechnology, China). Briefly, 5 × 10^7^ cells were harvested and washed with ice-cold PBS. The cells were resuspended in mitochondrial isolation reagent containing phenylmethylsulfonyl fluoride and protease inhibitors and homogenized in an ice-cold grinder. The homogenates were centrifuged at 600 ×*g* for 10 min and then the supernatants were further centrifuged at 11,000 ×*g* for 10 min at 4°C. The supernatants were decanted as the cytosolic fraction and the pellets were resuspended in 100 μl mitochondrial lysis buffer, which was kept as the mitochondrial fraction. The mitochondrial and cytosolic protein were subjected to western blotting. Tom20 served as a mitochondrial marker and α-GAPDH served as a cytosolic marker.

### Annexin V-FITC Assay

Apoptosis was determined by detecting phosphatidylserine (PS) exposure on cell membranes. Apoptotic cells were identified using AnnexinV-FITC Apoptosis Detection Kits (Beyotime, China) according to the manufacturer’s protocol. In brief, cells were challenged with SVV at a multiplicity of infection (MOI) of 1.0 or transfected with the indicated plasmids. After different lengths of time, the cells were washed once with PBS. Cells were then added to 195 μl of 1× Annexin V-FITC binding buffer, 5 μl of Annexin V-FITC and 10 μl of propidium iodide (PI) working solution for 15 min at RT in the dark. The cells were separated into three groups: live cells with little fluorescence, early apoptotic cells with green fluorescence, and necrotic and late-stage apoptotic cells showing both red and green fluorescence. All samples were observed using a fluorescence microscope (Nikon, Japan).

### Statistical Analysis

The various treatments were compared using an unpaired, two-tailed Student’s *t*-test assuming unequal variance. Treatment groups were compared with the control group, with significant differences indicated by ^∗^*P* < 0.05, ^∗∗^*P* < 0.01, and ^∗∗∗^*P* < 0.001. The experiments were repeated three times.

## Results

### SVV Infection Induces Apoptosis

**FIGURE 1:**
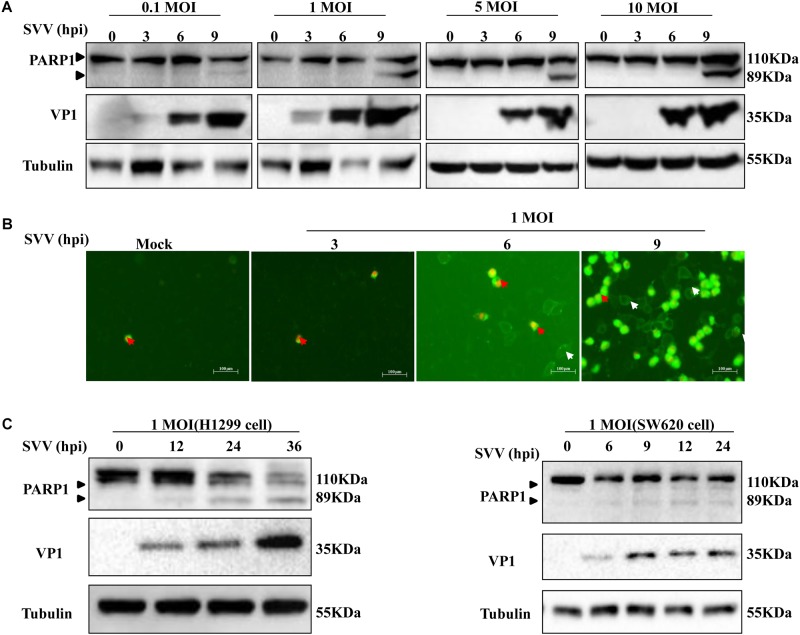
SVV induces cell apoptosis. **(A)** 293T cells were challenged with SVV at different MOIs (0.1, 1, 5, and 10), then the cells were harvested at indicated times and lysed, analyzed by western blotting with the indicated antibodies. **(B)** 293T cells were treated with SVV at an MOI for the indicated time, then the cells were stained with Annexin V-FITC/PI and observed with the fluorescence microscope. **(C)** H1299 cells and SW620 cells were infected with SVV at one MOI for the indicated time, and cleavage of PARP1 was determined by western blotting analysis.

Previously, it was shown that SVV-001 infection induced apoptosis (Yu et al., 2011). However, the underlying mechanisms remained elusive. In this study, we also observed morphological changes of SVV-infected 293T cells and the formation of CPE at 6 hours post-infection (hpi) (data not shown). To determine whether apoptosis occurred, expression of PARP1 was detected in SVV-infected cells showing CPE and cell death. PARP1, whose expression is triggered by activated caspase-3 in apoptotic cells, is a reliable biomarker of apoptosis (Oliver et al., 1998; Soldani and Scovassi, 2002). PARP1 and its cleavage fragments were detected by western blotting (Figure 1A). Levels of full-length PARP1 were significantly decreased at 9 hpi, while levels of cleaved PARP1 significantly increased when 293T cells were infected with SVV at higher MOIs. No cleaved PARP1 was detected in uninfected control cells.

Annexin V selectively binds to PS, and PS mainly distributes inside the cell membrane. During the early stage of apoptosis, PS rolls out onto the cell surface. Annexin-V positive cells were identified as early and late apoptosis populations. In order to further determine whether SVV-infected CPE was related to induction of apoptosis, we stained cells infected with SVV at a given different MOIs with DAPI and Annexin V/PI and calculated the ratio of apoptotic cells. Early apoptotic cells showed green fluorescence, and necrotic cells showed green and red fluorescence. The number of apoptotic cells showing green fluorescence increased over time, but was accompanied by few necrotic cells (Figure 1B). These results indicated that SVV infection induced 293T cell apoptosis.

Seneca Valley virus has been used as an oncolytic virus and is known to replicate in several tumor cell lines. H1299 cells and SW620 cells were challenged with SVV at a given MOI at the indicated times and showed similar results as for 293T cells. PARP1 was cleaved and the levels of cleaved PARP1 significantly increased in a time-dependent manner following SVV infection (Figure 1C). Together, these results showed that SVV can induce apoptosis.

### SVV Induces Apoptosis by Activating Caspase-3

**FIGURE 2:**
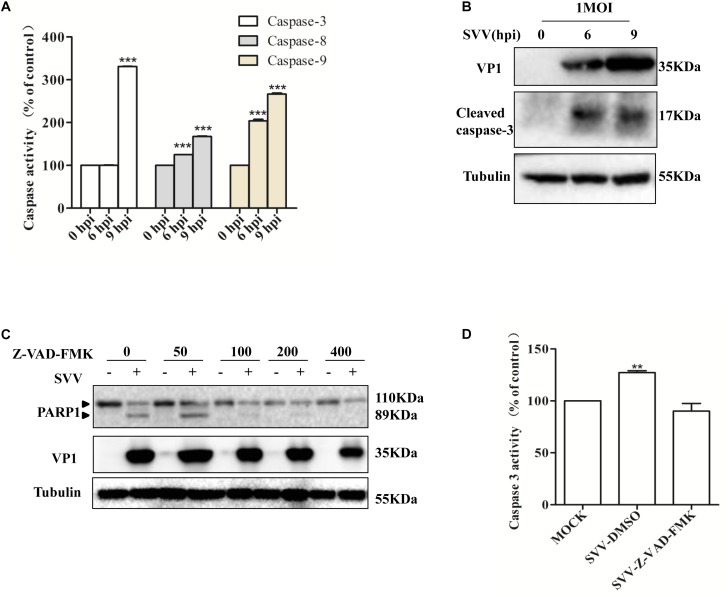
SVV induces cells apoptosis through both extrinsic and intrinsic pathways. **(A)** SVV infected 293T cells at an MOI for the indicated time, then the activities of the caspase-8, caspase-9, and caspase-3 were detected by the corresponding Caspase Activity Assay Kit. **(B)** SVV infected 293T cells at an MOI for the indicated time, then the cell lysates were analyzed by western blotting to detect the expression levels of cleaved caspase-3 bands. **(C)** Cell lysates from mock-treated or SVV-infected 293T cells in the presence of different concentrations of caspase inhibitor Z-VAD-FMK (0, 50, 100, 200, and 400 μM) were subjected to western blotting analysis with the indicated antibodies. **(D)** The activity of caspase-3 of the cells infected with SVV at one MOI in the absence or presence of Z-VAD-FMK at 50 μM by the Caspase-3 Activity Assay Kit (^∗^ 0.01 < *P* < 0.05; ^∗∗^ *P* < 0.01; ^∗∗∗^ *P* < 0.001).

Apoptosis can be initiated through the death receptor-mediated extrinsic pathway and the mitochondrial pathway (Schmitz et al., 2000; Wang, 2001; Pasquini et al., 2002). To gain insight into the mechanisms through which SVV induces apoptosis, we investigated caspase activity during SVV-induced apoptosis. 293T cells were infected with SVV at a MOI of 1.0 at the indicated time points and the activities of caspase-9, caspase-8 and caspase-3 were assessed with the corresponding caspase activity assay kit. Caspase-9, caspase-8 and caspase-3 were activated in a time-dependent manner following SVV infection, and their activities were markedly enhanced at 9 hpi in 293T cells compared with uninfected control cells (Figure 2A). We also found that cleavage of caspase-3 was observed after SVV infection by western blotting (Figure 2B).

To further confirm the involvement of caspase-3 in SVV-induced apoptosis, 293T cells were pre-treated with a broad-spectrum pan-caspase inhibitor (Z-VAD-FMK) at different concentrations and then infected with SVV. We found that cleavage of PARP1 was inhibited in the presence of increasing concentrations of Z-VAD-FMK (Figure 2C) and that the activity of caspase-3 was significantly inhibited in the presence of Z-VAD-FMK (Figure 2D). Taken together, these results suggested that SVV can initiate apoptosis through intrinsic and extrinsic pathways.

### SVV 2C and 3C^pro^ Induce Apoptosis by Activating Caspase-3

**FIGURE 3:**
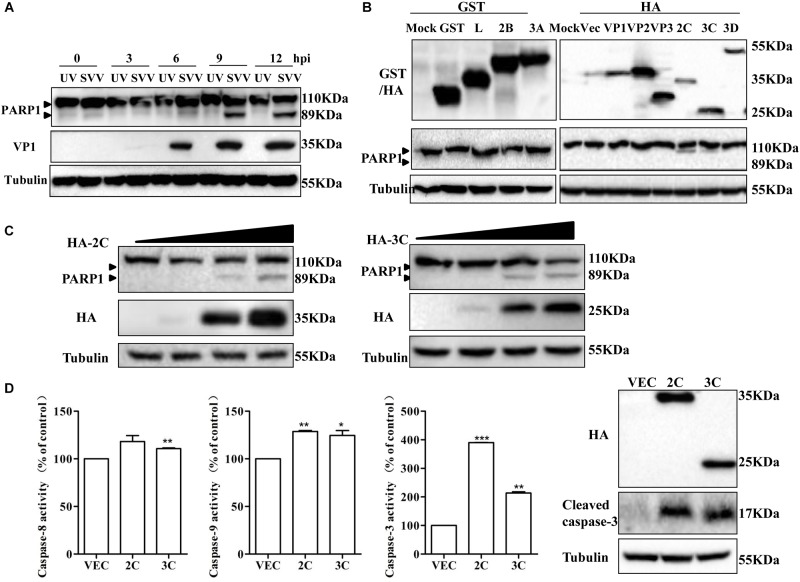
SVV 2C and 3C^pro^ induce 293T cells apoptosis via activating caspase-3. **(A)** SVV were inactivated with UV, then 293T cells were challenged with the inactivated SVV and SVV at one MOI at the indicated time points post-infection, the cells were collected and lysed to be subjected to western blotting with the indicated antibodies. **(B)** 293T cells were transfected with the indicated plasmids expressing GST-tag or HA-tag viral proteins or the corresponding empty vector plasmids. The expression of these viral proteins and cleavage of PARP1 were detected by western blotting at 24 hpt. **(C)** 293T cells were transfected with different doses of HA-2C or HA-3C expressing plasmids for 24 h. The expression of corresponding proteins was determined by western blotting. **(D)** 293T cells were transfected with the indicated plasmids expressing 2C or 3C proteins, then the cells were harvested to measured the activities of the caspase-8, caspase-9 and caspase-3 by the corresponding Caspase Activity Assay Kit. The cell lysates were analyzed by western blotting to detect the expression levels of cleaved caspase-3 bands (^∗^0.01 < *P* < 0.05; ^∗∗^*P* < 0.01; ^∗∗∗^*P* < 0.001).

To determine if SVV-induced apoptosis was related to viral replication, 293T cells were infected with SVV or UV-inactivated SVV. Changes in PARP1 expression were assessed at 12 hpi. UV-inactivated SVV could not induce cleavage of PARP1 (Figure 3A), indicating that SVV-induced apoptosis depended on SVV replication in cells. To identify which viral proteins were responsible for SVV-induced apoptosis, 293T cells were transfected with plasmids encoding viral proteins and the abundance of PARP1 was assessed by western blotting at 24 h posttransfection. Levels of cleaved PARP1 were significantly increased in the presence of 2C and 3C^pro^, whereas no cleaved PARP1 were detected in cells expressing other viral proteins (Figure 3B). We therefore explored whether 2C and 3C^pro^ were responsible for inducing apoptosis. 293T cells were transfected with increasing doses of HA-2C or HA-3C. After 24 h, PARP1 abundance was assessed, and the results showed that 2C and 3Cpro could induce cleavage of PARP1 and induce apoptosis (Figure 3C). Morphological changes of 293T after transfection with 2C and 3C^pro^ were observed by fluorescence microscopy after Annexin V FITC/PI staining (Supplementary Figure S1). We found that the frequency of Annexin V-positive cells increased in cells expressing increasing amounts of 2C protein, suggesting that apoptotic cells were increased in a 2C-dose-dependent manner. These results demonstrated that SVV 2C and 3C^pro^ proteins were responsible for inducing 293T cell apoptosis.

Caspases are crucial for execution of apoptosis, and apoptosis is initiated due to their activation (Stegh and Peter, 2001). 293T cells were transfected with plasmids encoding HA-2C, HA-3C or an empty vector and 24 h later, the activities of caspase-3, caspase-8 and caspase-9 were measured using the corresponding caspase activity assay kit (Figure 3D). Meanwhile, cleavage of caspase-3 was observed after transfection of 2C and 3C^pro^ by western blotting (Figure 3D). SVV 2C protein significantly increased the activity of caspase-3 and caspase-9 and 3C^pro^ protein activated caspase-3, caspase-8 and caspase-9. Activation of caspase-3 and caspase-9 is a key irreversible point in mitochondrion-mediated apoptosis. In the death receptor-mediated pathway, caspase-8 is activated downstream of the death inducing signaling complex, which then results in downstream activation of caspase-3 and initiation of apoptosis. These results revealed that SVV 2C protein induced apoptosis by the mitochondrion-mediated intrinsic pathway, while 3C^pro^ probably induced apoptosis through both the mitochondrial pathway and the extrinsic death receptor pathway.

### 2C Protein Is Located in Mitochondria and Interacts With Bcl-X_L_

**FIGURE 4:**
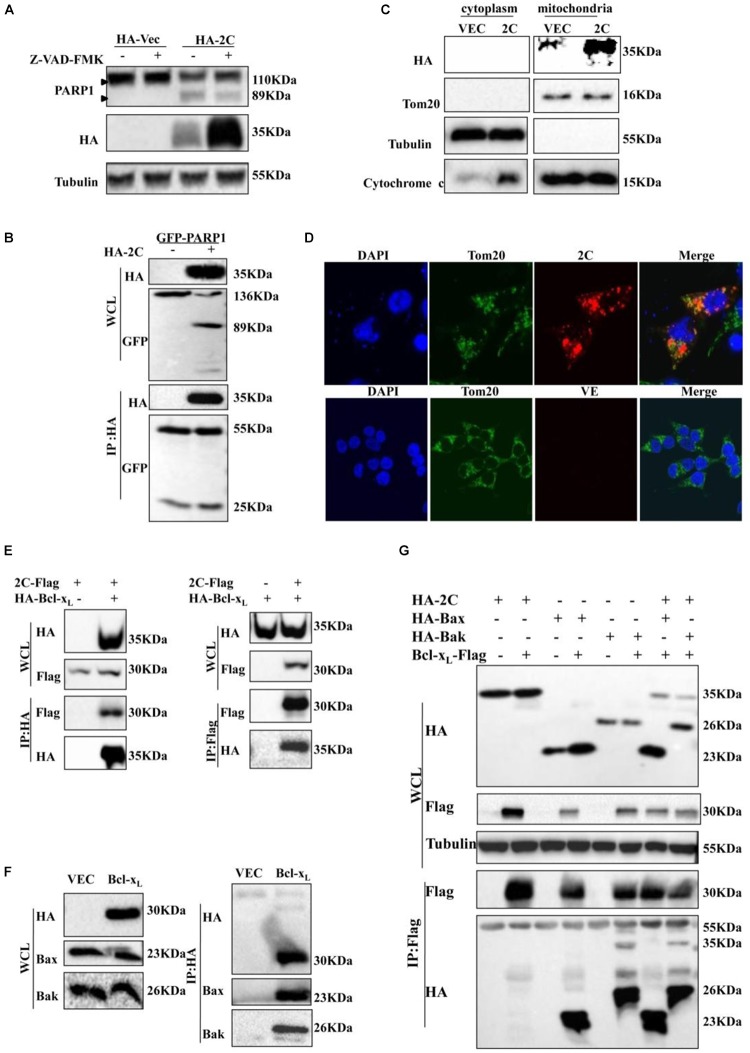
2C protein is located in the mitochondria and interacts with Bcl-x_L_. **(A)** 293T cells were transfected with HA-2C in the absence or presence of Z-VAD-FMK (50 μM) for 24 h. The cells lysates were analyzed by western blotting to detect the expression levels of cleaved PARP1 bands. **(B)** 293T cells were cotransfected with HA-2C and GFP-PARP1 for 24 h and then subjected to immunoprecipitation (IP) with anti-HA antibody. The immunoprecipitates were analyzed by western blotting with the indicated antibodies. **(C)** Western blotting analysis of 2C protein in the cytosol and mitochondrial fractions of cells expressing 2C protein. Cytosolic and mitochondrial fractions were separated, and equal amounts of proteins from each fraction were immunoblotted with anti-GAPDH, anti-cytochrome C, anti-HA, or anti-Tom20 antibody (internal control of mitochondrial fraction). **(D)** An experiment indicating that 2C was located in the mitochondria. Cells were transfected with HA-2C (red arrow) or empty vector. At 24 h posttransfection, the cells were fixed and analyzed by confocal fluorescence microscopy. Tom20 served as a mitochondrial marker and was stained with anti-Tom20 (green) antibody, and then imaged by confocal microscopy. **(E)** 293T cells were cotransfected with HA-Bcl-x_L_ and 2C-Flag for 24 h. Lysates were immunoprecipitated by anti-HA or anti-Flag antibody as indicated. **(F)** 293T cells were transfected with HA-Bcl-x_L_ for 24 h and then subjected to immunoprecipitation with anti-HA, anti-Bak, or anti-Bax antibody. **(G)** The indicated plasmids were cotransfected into 293T cells for 24 h and analyzed by Co-IP using the indicated antibody.

The 2C protein of picornaviruses is multifunctional during the virus life cycle and is highly conserved (Hales et al., 2008). SVV 2C protein can induce apoptosis by directly cleaving PARP1, but cleavage of PARP1 was significantly reduced in the presence of Z-VAD-FMK (Figure 4A). We confirmed that 2C protein did not interact with PARP1 by Co-IP, indicating that 2C did not directly cleave PARP1 to induce apoptosis (Figure 4B).

2C induced-apoptosis was associated with the mitochondrion-mediated intrinsic pathway and mitochondria are initiators and transducers of apoptosis (Green and Reed, 1998; Li and Li, 2000). To further investigate the role of mitochondria in the induction of apoptosis by the SVV 2C protein, we assessed the subcellular distribution of 2C protein. 293T cells were transfected with a plasmid encoding HA-2C or empty vector, and both cytosolic and mitochondrial fractions were isolated and analyzed by western blotting. 2C protein was only in detected the mitochondrial fraction, with undetectable levels in the cytosol (Figure 4C). 2C promoted cytochrome C release into the cytoplasm. To further assess whether 2C protein was solely located in mitochondria, we carried out confocal microscopy analysis. Confocal microscopy analysis revealed an extensive overlap between the distribution of 2C protein and Tom20 (Figure 4D), a marker of mitochondria. These results indicated that the 2C protein co-localized with Tom20 and solely localized to the mitochondria.

**FIGURE 5:**
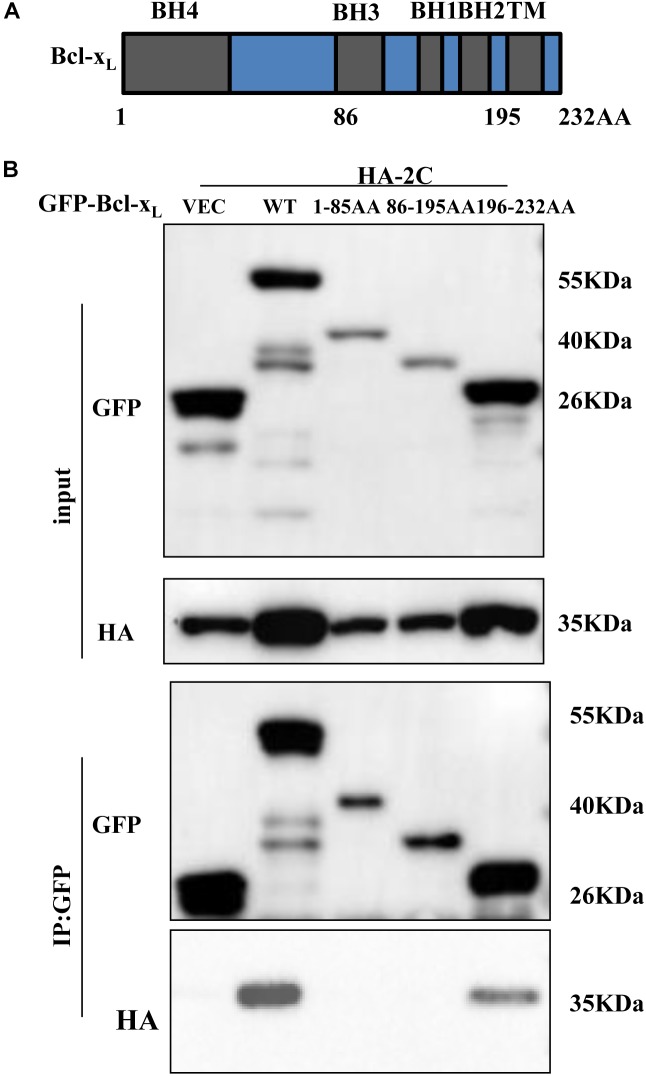
2C protein interacts with the C-terminal region (196-232AA) of Bcl-x_L_ protein. **(A)** Schematic diagram of Bcl-x_L_. BH, Bcl-2 homology domain; TM, transmembrane domain. **(B)** GFP-Bcl-x_L_ or its truncate mutants and HA-2C were individually transfected into 293T cells. The cell lysates were immunoprecipitated with an anti-GFP antibody and then immunoblotted with the indicated antibodies.

Next, we investigated whether 2C-induced cell apoptosis occurred via interaction with members of Bcl-2 family proteins, which are located in mitochondria and regulate their function. 293T cells were transfected with a plasmid encoding HA-tagged 2C protein and IP experiments were conducted using an anti-HA antibody. We found that the 2C protein interacted with the anti-apoptotic proteins, Bcl-2 and Bcl-x_L_ (Supplementary Figure S2A). We further determined that 2C protein only interacted with Bcl-x_L_ by co-IP (Figure 4E and Supplementary Figure S2B). 2C protein interacted with endogenous Bcl-x_L_ as well (Supplementary Figure S2C). Furthermore, since the anti-apoptotic protein Bcl-x_L_ can interact with the pro-apoptotic proteins (Figure 4F), Bax and Bak, it remained unclear whether 2C disturbed this balance. 293T cells were cotransfected with the indicated plasmids in the presence or absence of 2C protein and subjected to a Co-IP assay (Figure 4G). The results indicated that 2C protein did not disrupt the interaction of Bcl-x_L_ with Bax or Bak and did not disturb the balance among these proteins. Taken together, these data demonstrated that 2C was located in the mitochondria, interacted with Bcl-x_L_, but did not disturb the balance between anti-apoptotic and pro-apoptotic proteins, indicating the region of interaction between Bcl-x_L_ and 2C differed from that between Bcl-x_L_ and Bak or Bax. To validate this hypothesis, different truncates of the Bcl-x_L_ gene were constructed (Figure 5A), and CO-IP experiments showed that the C-terminal region (196-232AA) of the Bcl-x_L_ protein interacted with the 2C protein (Figure 5B).

### 3C^pro^ Induction of Apoptosis Depended on Its Protease Activity

**FIGURE 6:**
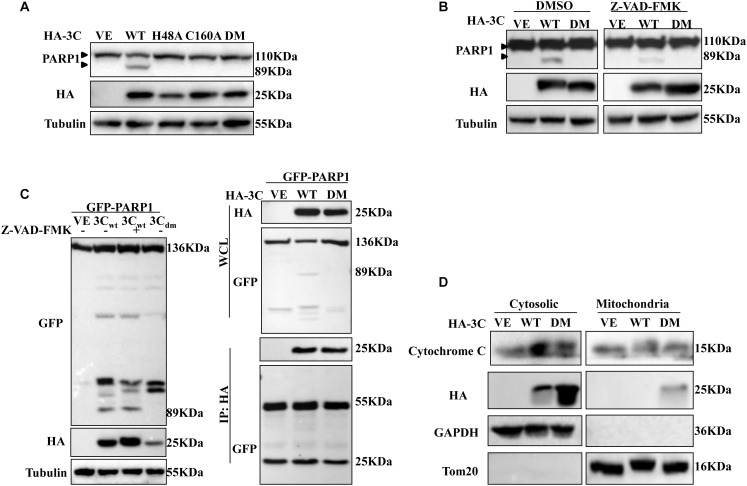
3C^pro^ induces apoptosis dependent on its enzyme activity. **(A)** 293T cells were transfected with HA-3C-WT, HA-3C-H48A, HA-3C-C160A, HA-3C-DM or empty vector, respectively, for 24 h. Then the cell lysates were analyzed by western blotting to detect the expression levels of cleaved PARP1 bands. **(B)** 293T cells were transfected with the indicated constructs in the presence or absence of Z-VAD-FMK (50 μM). Cell lysates were collected for western blotting analysis with the indicated antibodies. **(C)** 293T cells were cotransfected with HA-3C or HA-3C-DM and GFP-PARP1 in the presence of Z-VAD-FMK (50 μM) for 24 h to the cleaved PARP1 using anti-GFP antibody. Co-IP was performed with the indicated antibodies. Samples of both cell lysates and immunoprecipitates were subjected to western blotting with mouse anti-GFP and mouse anti-HA antibodies. The immunoprecipitates were analyzed by immunoblotting with the indicated antibodies. **(D)** Western blotting analysis of 3C protein in the cytosol and mitochondrial fractions of cells transfected with HA-3C or HA-3C-DM or empty vector. Cytosolic and mitochondrial fractions were separated and detected with the indicated antibodies.

3C^pro^ can cleave many important adaptors using its protease activity. Thus, we considered that whether 3C^pro^-induced apoptosis was associated with its protease activity. 293T cells were transfected with plasmid encoding either 3C^wt^ protein, the single-site 3C mutants H48A or C160A, or the double-mutant 3C_dm_ protein (H48A/C160A), and the abundance of PARP1 was assessed by western blotting (Figure 6A). 3C^pro^ mutants with defective protease activity lost their ability to mediate cleavage of PARP1, and morphological changes were not observed in apoptotic cells after Annexin V/PI staining and protease inactivation (Supplementary Figure S1). Although 3C^pro^ can activate caspase-3, activation was significantly inhibited in the presence of Z-VAD-FMK (Figure 6B). When 293T cells were cotransfected with plasmids encoding 3C^pro^ and PARP1 protein in the absence or presence of Z-VAD-FMK, we found that 3C^pro^ did not directly cleave or interact with PARP1 (Figure 6C).

We also determined the subcellular distribution of 3C^pro^. 3C^pro^ from both cytosolic and mitochondrial fractions was prepared and analyzed by western blotting. 3C^pro^ induced cytochrome C release into the cytoplasm and was itself mainly distributed in the cytoplasm. However, some 3C^pro^ trafficked to mitochondria after sites responsible for its protease activity were mutated (Figure 6D). Collectively, these results showed that 3C^pro^-induced apoptosis depended on its protease activity.

## Discussion

The persistent occurrence of SVV infection in different regions suggests a potential risk of pandemic outbreak (Pasma et al., 2008; Hause et al., 2016; Leme et al., 2017; Zhu et al., 2017). Previous studies reported that SVV selectively infected cancer cells with neuroendocrine features, including a subset of small-cell lung cancers and pediatric neuroendocrine solid tumors (Seshidhar et al., 2007). Moreover, SVV has been used as an oncolytic virus to treat human neuroendocrine cancers (Rudin et al., 2011; Burke et al., 2015; Coffin, 2015). However, the detailed mechanisms through which SVV induces apoptosis and kills tumor cells are still unclear.

In this study, we demonstrated that SVV induces CPE in 293T cells and induces apoptosis in multiple cancer cell lines. These findings are consistent with previous studies (Seshidhar et al., 2007; Yu et al., 2011). Apoptosis can be triggered either by an extrinsic death receptor stimulus or through intrinsic mitochondria-mediated signaling. Although both pathways engage the same proteolytic caspase cascade and interface at the point of downstream executioner caspase-3 activation, initiator caspase-8 and caspase-9 discriminate between the extrinsic and intrinsic pathways, respectively. Then the downstream effector caspase-3 was proteolytically activated to induce the execution phase of apoptosis (Clarke and Tyler, 2009; Galluzzi et al., 2012). We confirmed that SVV induced apoptosis through extrinsic and intrinsic pathways by activating caspase-8 and caspase-9. Caspase-3 plays a very important role in both pathways, and activity of caspase-3 induced by SVV infection was inhibited in the presence of Z-VAD-FMK, suggesting that apoptosis induced by SVV was not entirely dependent on caspase-3 activation. SVV can also induce autophagy (Yu et al., 2011), suggesting that SVV uses multiple strategies to induce cell death and boost its replication.

We found that both SVV 2C and 3C^pro^ proteins were involved in triggering apoptosis. These two proteins caused cleavage of PARP1 in a dose-dependent manner and increased the number of apoptotic cells after Annexin V/PI staining. The 2C protein induced apoptosis through activating caspase-9 and caspase-3, suggesting that 2C induced apoptosis through the mitochondrion-mediated intrinsic pathway. The Bcl-2 family members regulate mitochondrion-mediated apoptosis by directly controlling mitochondrial membrane permeability and release of apoptotic factors into the cytoplasm (Yang and Korsmeyer, 1996; Crompton, 2003). The Bcl-2 family is composed of pro-apoptotic and anti-apoptotic members (Gross et al., 1999). In normal cells, the anti-apoptotic protein Bcl-2 promotes cell survival by binding to the pro-apoptotic proteins Bak and Bax. The pro-apoptotic proteins Bax and Bak are crucial for the release of cytochrome C from mitochondria (Wei et al., 2001; van Loo et al., 2002). When the balance of the major apoptosis regulators is disrupted, apoptosis is initiated (Antonsson, 2004). We found SVV 2C protein was solely localized in mitochondria and triggered release of cytochrome C into the cytoplasm (Figure 4C). This represented a different phenotype compared with other picornavirus 2C proteins. We also found that the 2C protein interacted with the anti-apoptotic protein Bcl-x_L_, but did not disrupt the balance of major apoptosis regulators, indicating that the region of interaction between 2C and Bcl-x_L_ differs from that between Bcl-x_L_ and Bak or Bax. The BH1, BH2, and BH3 domains of multi domain anti-apoptotic and pro-apoptotic members can form a hydrophobic binding groove, and Bax or Bak retrotranslocation from the mitochondria requires recognition of its exposed BH3 motif by the hydrophobic groove of Bcl-x_L_ and interaction between the C-terminal Bcl-x_L_ helix and Bax or Bak (Edlich et al., 2011; Todt et al., 2013; Jeng et al., 2018). Bcl-x_L_ and Bax interact on mitochondria, retrotranslocate together, and dissociate in the cytosol (Edlich et al., 2011). We found that 2C protein interacted with the C-terminal region (196-232AA) of Bcl-x_L_ protein, which interfered with the interaction between Bcl-x_L_ and Bax and increased mitochondrial levels of endogenous Bax. Future studies will help to determine whether this hypothesis is correct.

The picornavirus 2C protein is a helicase-like polypeptide involved in RNA synthesis and contains nucleoside triphosphate-binding and helicase motifs (Gorbalenya et al., 1990). The structures of 2C proteins of different picornavirus genera differ, making interpretation of their specific functions challenging (Chunling et al., 2012; Guan et al., 2017; Guan et al., 2018). Amino acid sequence analyses revealed that SVV 2C, a hydrophobic protein, does not possess a transmembrane region^1^, which makes it different from other 2C picornavirus proteins (Guan et al., 2017; Guan et al., 2018). In addition, we found the N-terminal (1-102AA) of 2C was essential for inducing apoptosis (not shown). Our investigation also showed that 2C induced apoptosis by targeting mitochondria and causing release of cytochrome C into the cytoplasm. Further work will be required to analyze 2C protein structure to identify the key checkpoint. In this study, we found that 3C^pro^ could activate caspase-9, caspase-8 and caspase-3, indicating 3C^pro^ triggered apoptosis through both the mitochondrial pathway and the death receptor pathway. 3C^pro^ plays a crucial role in the viral life cycle and virus-host interactions, and its protease activity is indispensable (Qian et al., 2017). We confirmed that 3C^pro^-induced apoptosis was dependent on its protease activity, as 3C^pro^ mutants with impaired protease activity could not induce apoptosis. SVV 3C protease activity plays a vital role in cleavage of viral polyprotein and host proteins. Previous studies have reported that 3C^pro^ cleaved the host adaptor molecules MAVS, TRIF, and TANK to inhibit host innate immune responses (Qian et al., 2017). Ectopic expression of MAVS and TRIF potently stimulated intrinsic apoptotic machinery to induce cell death (Kaiser and Offermann, 2005; Yu et al., 2009). Our studies indicated that 3C^pro^-induced apoptosis was similar to that of CVB3 3C^pro^ (Mukherjee et al., 2011). We found SVV 3C^pro^ was solely localized in the cytoplasm, however, part of 3C-DM distributed in the mitochondria, which may be related to the proteolytic activity of 3C^pro^. And 3C-DM could interact with the host adaptor molecules MAVS (Qian et al., 2017), which is localized in the mitochondria (Seth et al., 2005). Further studies on the mechanisms of 3C^pro^-induced apoptosis need to be carried out.

In this study, we demonstrated that SVV may kill tumor cells by inducing apoptosis, and that 2C and 3C^pro^ proteins were involved in this process. These findings imply that SVV 2C and 3C^pro^ may represent virulence factors that help SVV replicate in tumor cells. Moreover, we can control and limit the spread of this virus and improve livestock safety. SVV may represent a safe, effective and potential biological therapy for cancer treatment.

## Author Contributions

PQ, HC, and XL designed the research, provided funding, integrated the data, proofread and revised the manuscript. TL, MW, and LQ designed and performed the research, collected and analyzed the data, and drafted and revised the manuscript. TL wrote the manuscript. All authors read and approved the final version of the manuscript to be published.

## Conflict of Interest Statement

The authors declare that the research was conducted in the absence of any commercial or financial relationships that could be construed as a potential conflict of interest.

## Footnotes

1 http://www.cbs.dtu.dk/services/TMHMM

## Supplementary Material

FIGURE S1 2C and 3C^pro^ induce apoptosis by Annexin V-FITC assay. 293T cells were grown in 24-well plates, and the monolayer cells were transfected with different doses plasmids of HA-2C or HA-3C-WT, HA-3C-H48A, HA-3C-C160A, HA-3C-DM or empty vector, respectively, for 24 h, then the cells were stained with AnnexinV-FITC/PI and observed with the fluorescence microscope. White arrow: Apoptosis cells; Red arrow: Necrosis cells.

FIGURE S2 2C protein interacts with endogenous Bcl-x_L_. **(A)** 293T cells were transfected with HA-2C for 24 h and then subjected to immunoprecipitation (IP) with anti-HA antibody. The immunoprecipitates were analyzed by western blotting with the indicated antibodies. **(B)** 293T cells were cotransfected with HA-2C and Flag-Bcl-2 for 24 h. Lysates were immunoprecipitated by anti-HA or anti-Flag antibody as indicated. **(C)** 293T cells were transfected with HA-2C for 24 h and then subjected to immunoprecipitation with anti-HA antibody. The immuno- precipitates were analyzed by western blotting with the indicated antibodies.
