# Supplementary figures and images for: Seneca Valley Virus 2C and 3Cpro Induce Apoptosis via Mitochondrion-Mediated Intrinsic Pathway
Source: Front Microbiol. 2019 May 29;10:1202. doi: 10.3389/fmicb.2019.01202 (PMC6549803; doi:10.3389/fmicb.2019.01202)

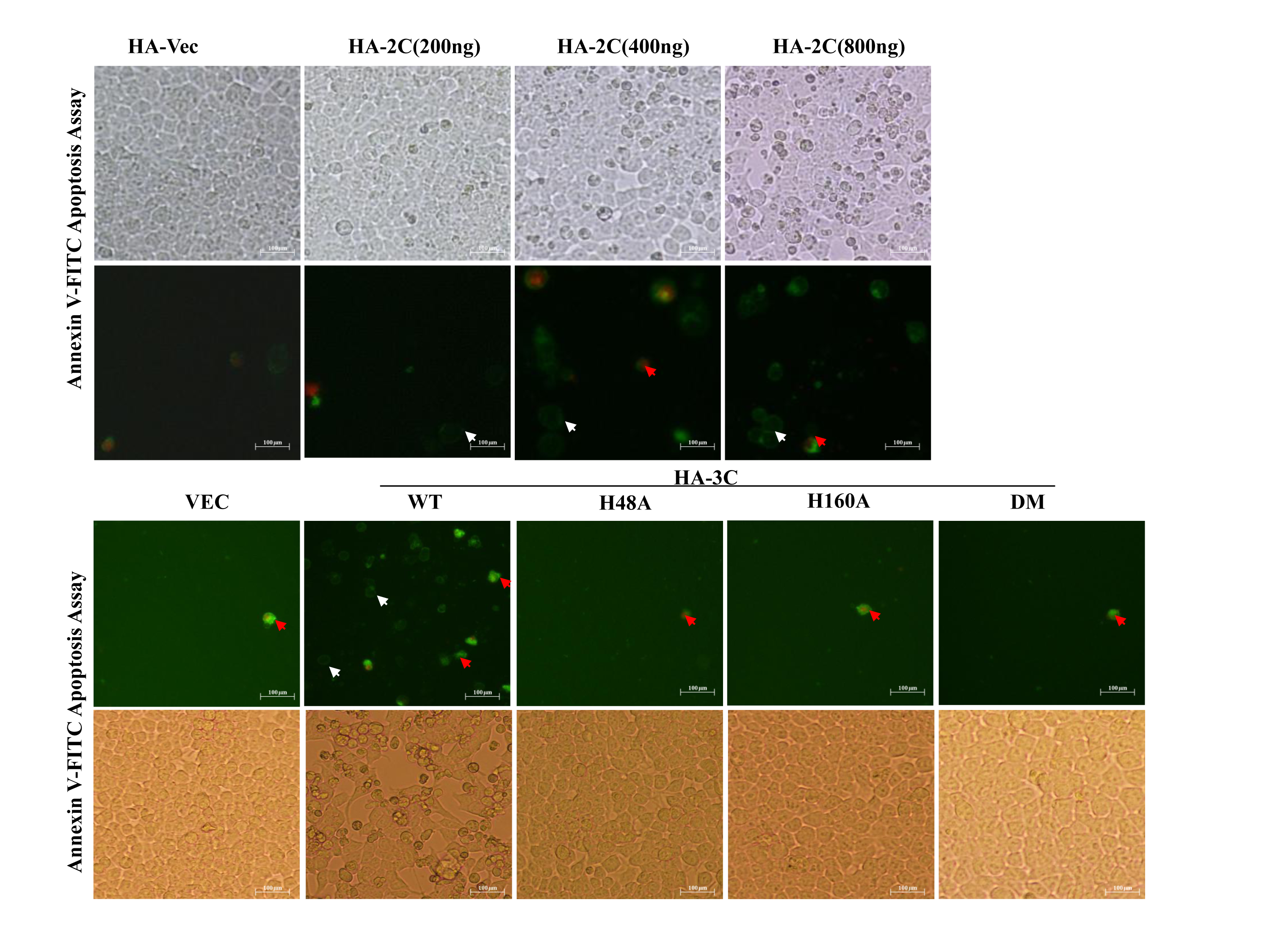

Supplement: FIGURE S1 — 2C and 3Cpro induce apoptosis by Annexin V-FITC assay. 293T cells were grown in 24-well plates, and the monolayer cells were transfected with different doses plasmids of HA-2C or HA-3C-WT, HA-3C-H48A, HA-3C-C160A, HA-3C-DM or empty vector, respectively, for 24 h, then the cells were stained with AnnexinV-FITC/PI and observed with the fluorescence microscope. White arrow: Apoptosis cells; Red arrow: Necrosis cells. [file Image_1.TIF]

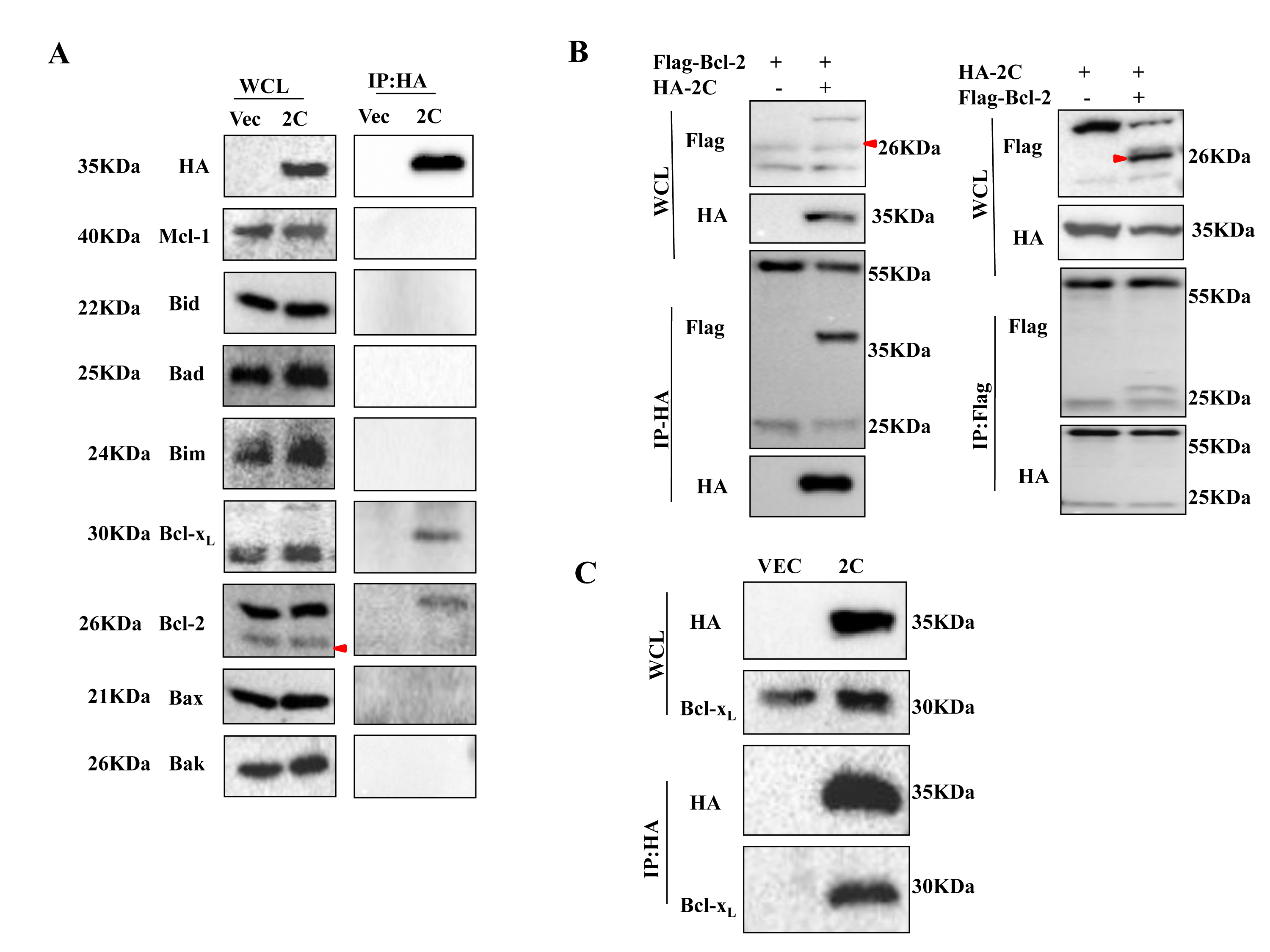

Supplement: FIGURE S2 — 2C protein interacts with endogenous Bcl-xL. (A) 293T cells were transfected with HA-2C for 24 h and then subjected to immunoprecipitation (IP) with anti-HA antibody. The immunoprecipitates were analyzed by western blotting with the indicated antibodies. (B) 293T cells were cotransfected with HA-2C and Flag-Bcl-2 for 24 h. Lysates were immunoprecipitated by anti-HA or anti-Flag antibody as indicated. (C) 293T cells were transfected with HA-2C for 24 h and then subjected to immunoprecipitation with anti-HA antibody. The immuno- precipitates were analyzed by western blotting with the indicated antibodies. [file Image_2.tif]
